# A Novel Function of RING Finger Protein 10 in Transcriptional Regulation of the Myelin-Associated Glycoprotein Gene and Myelin Formation in Schwann Cells

**DOI:** 10.1371/journal.pone.0003464

**Published:** 2008-10-21

**Authors:** Shinya Hoshikawa, Toru Ogata, Sayaka Fujiwara, Kozo Nakamura, Sakae Tanaka

**Affiliations:** 1 Department of Orthopaedic Surgery, Faculty of Medicine, The University of Tokyo, Bunkyo-ku, Tokyo, Japan; 2 Division of Motor Dysfunction, Research Institute, National Rehabilitation Center, Tokorozawa, Saitama, Japan; Medical College of Georgia, United States of America

## Abstract

Myelin-associated glycoprotein (MAG) has been detected in Schwann cells prior to the onset of myelination, suggesting its functions in the initiation of myelination. However, transcriptional regulatory mechanisms of MAG remain to be elucidated. Here, we analyzed the promoter of the *MAG* gene by using luciferase reporter systems in the primary rat Schwann cells. We identified a novel *cis*-acting element located 160 bp upstream from the *MAG* transcription initiation site. Using the identified *cis*-element as a bait, we performed yeast one-hybrid screening and isolated a cDNA encoding a *RNF10* as a putative trans-acting protein. When overexpressed in Schwann cells, RNF10 enhanced the activity of the *MAG* promoter. When RNF10 expression in Schwann cells was knocked down by siRNA, endogenous MAG mRNA and protein expression decreased. Furthermore, we evaluated myelin synthesis using Schwann cell-DRG neuron cocultures. When Schwann cells were infected with retrovirus expressing RNF10 siRNA, myelin formation was inhibited. These data suggest that RNF10 regulates MAG expression and is required for myelin formation.

## Introduction

Myelin sheath is a unique component of the nervous system. It ensheaths the axons in the peripheral nervous system (PNS) and central nervous system (CNS) of vertebrates. It increases axonal conduction velocity, thereby allowing saltatory conduction [1]. Given the functional importance of myelin, it is of particular interest to clarify the molecular mechanisms underlying the differentiation of myelinating glial cells and formation and maintenance of myelin. It is widely accepted that several molecules expressed on axons, such as neuregulin [2], convey axonal signals that trigger myelin-forming cells, namely, Schwann cells in the PNS, to initiate the process of myelination. Therefore, molecules expressed at the axon-glia junction are also likely to be involved in the onset of myelination. Among several myelin-membrane proteins, only myelin-associated glycoprotein (MAG) appears in the periaxonal area of the Schwann cell [3], [4].

The MAG expression is induced when a 1∶1 relationship is established between Schwann cells and axons; such a relationship facilitates the contact of the processes of each Schwann cell to the axon at the promyelinating Schwann cell stage. MAG has been detected on Schwann cell surface prior to the onset of myelination [5] and has long been thought to be involved in glia-axon interactions [6]. Previous reports have shown that MAG performs important positive functions in the differentiation of myelinating glial cells, glia-axon interactions, and myelination [7]. Therefore, MAG is believed to play an important role in the onset of myelination, and the regulatory mechanisms underlying MAG expression may be important in the initiation of myelination processes. Along this line, our previous study revealed that activation of the PI3K pathway induced MAG expression simultaneously with Schwann cell differentiation [8]. However, the transcriptional regulatory mechanisms underlying MAG expression remain poorly understood. In the present study, we investigated the regulatory mechanisms of Schwann cell myelination through analyzing the regulation of MAG expression at the transcriptional level.

The *MAG* promoter contains neither the canonical TATA box nor initiator motifs [9]. The specific *cis*-elements and the transcriptional regulatory factors remain to be identified [10]. Since the expression of MAG is closely related to the onset of myelination, transcriptional regulation of the *MAG* gene appears to have a strong influence on the mechanism underlying the initiation of myelination. Therefore, in this study, we specifically aimed to identify the positive *cis*-acting element in the *MAG* gene and the specific transcriptional regulatory factor in Schwann cells, with the ultimate goal to elucidate the regulatory mechanisms that control the gene activity in differentiating Schwann cells.

## Results

### The −162/−143 Region is Essential for *MAG* Promoter Activity

**Figure 1:**
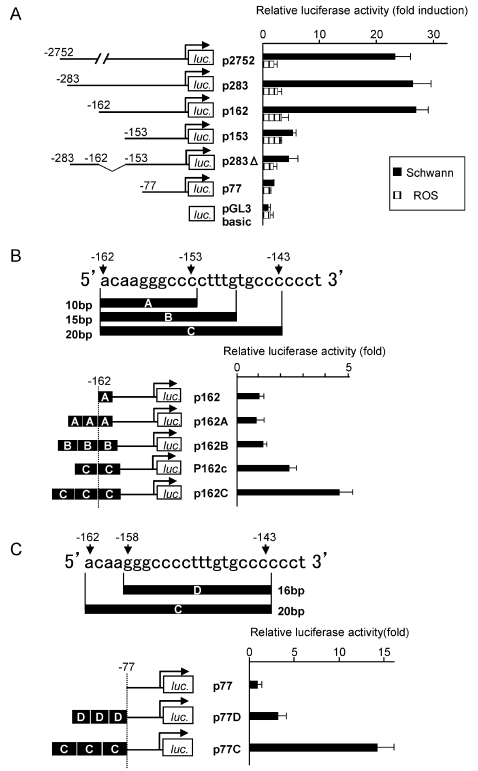
Luciferase activity of Schwann cells transiently transfected with a *MAG* promoter construct. (A) Transient cotransfections of Schwann cells and ROS cells using various rat *MAG* reporter constructs. The largest *MAG*-luciferase reporter plasmid had a 2.7-kb promoter. Numerous sequences with 5′ deletion were constructed, including reporter plasmids containing 283-, 162-, 153-, and 77-bp segments of the MAG promoter. A sequence with internal deletion of a segment from −162 to −153 was also constructed. The observed firefly luciferase activity is normalized with the Renilla luciferase activity and the results are expressed as fold induction compared with empty vector in Schwann cells. Deletion of a region between −162 and −153 greatly reduced the luciferase activity. In contrast, the luciferase activity showed subtle changes in the ROS cell. (B, C) To examine the positive effects of the sequence between −162 and −153 on the promoter activity, we generated and analyzed various lengths of tandem repeats downstream from −162 (B) and upstream from −143 (C). The normalized luciferase activity is expressed as fold induction compared with the value of either p162 in (B) or p77 in (C) respectively. Only the reporter constructs bearing 20-bp tandem repeats (−162 to −143) could increase the luciferase activity in a repeat number-dependent manner. Therefore, we believe that this 20-bp sequence (5′-ACAAGGGCCCCTTTGTGCCC-3′) is required and sufficient for the activation of the *MAG* promoter and that it is a *cis*-acting element.

To identify the *cis*-element(s) required for the promoter activity of the rat *MAG* gene, serial and internal deletion luciferase reporter constructs were analyzed in primary cultured rat Schwann cells by using transient transfection assays. The promoter region of the *MAG* gene obtained from rat genomic DNA was cloned into a pGL3-Basic luciferase reporter vector. To measure the basal activity, an empty pGL3-Basic vector was used as a control vector. The reporter construct p2752, containing rat *MAG* promoter region extending from −2752 to +78, was adequate for the detection of reporter gene expression in Schwann cells. Sequential deletion of the region between −2752 and −162 in the rat *MAG* promoter caused a modest increase in the promoter activity. However, further deletion of a 9-nucleotide stretch (−162 to −153) resulted in a marked decrease in the reporter gene activity in Schwann cells. Deletion of the sequence between −162 and −153 in the p283 construct (p283Δ) reduced the luciferase expression level, which was then similar to the level in p153. These data suggested that a positive regulatory element exists within the region from −162 to −153. The deletion analysis of the *MAG* promoter revealed the induction of luciferase activity in Schwann cells but much less in rat osteosarcoma (ROS) cells. Therefore, the *MAG* promoter would contain a Schwann cell-specific *cis*-acting element (Fig 1A).

To examine whether a 10-bp sequence between −162 and −153 is adequate to function as a *cis*-element, a 10-, 15-, or 20-bp tandem repeat sequence downstream from position −162 was appended to the 5′ end of the *MAG* promoter sequence of p162, thereby generating p162A, p162B, and p162C constructs, respectively (Fig 1B), as previously described [11]. Only the construct containing the 20-bp (−162 to −143) tandem repeats (p162c/C) could increase the luciferase activity. The triple repeat of the 20 bp (p162C) increased luciferase activity than the double repeat (p162c) (Fig 1B).

The deletion construct p77 exhibited a low luciferase activity, and this activity was induced only by the core promoter (−25 to +40) [12]. To examine the importance of the 5′ end of the 20-bp sequence, a 16- or 20-bp tandem repeat sequence upstream from position −143 was appended to the 5′ end of the minimal *MAG* promoter sequence p77, thereby generating p77D and p77C constructs, respectively (Fig 1C). Identical to the abovementioned observation, only the construct containing 20-bp tandem repeats (p77C) could markedly increase the luciferase activity (Fig 1C). Therefore, we believe that this 20-bp sequence (−162 to −143: 5′- ACAAGGGCCCCTTTGTGCCC -3′) is essential for the activation of transcription. We designated this sequence as “Schwann cell-specific element (SSE).” This sequence is completely conserved among the rat, human, and mouse (Table 1).

**Table 1:** Comparison of the promoter region of the MAG gene.

RAT:	aga------------gctgcccagcctcagccctagccctgtg
MOUSE:	…cccaggaatcaa………t.c…………ga‥
HUMAN:	.a.cccaggcgtcag………t.cg…‥c------…
RAT:	gtg**acaagggcccctttgtgccc**ccctcccccgggggcaggga
MOUSE:	…………………………………….
HUMAN:	……………………………….a…‥

Sequence alignments of the MAG promoter region. Identities are indicated by dots and gaps by dashes. Bold characters show the 20 bp MAG promoter cis-element (−162/−143), designated “SSE”. SSE sequence is completely conserved between Rat, Mouse and Human.

### Isolation of Transcriptional Regulatory Factor RNF10 that Interacts with the 20-bp *Cis*-acting Element Region in the *MAG* Promoter

**Figure 2:**
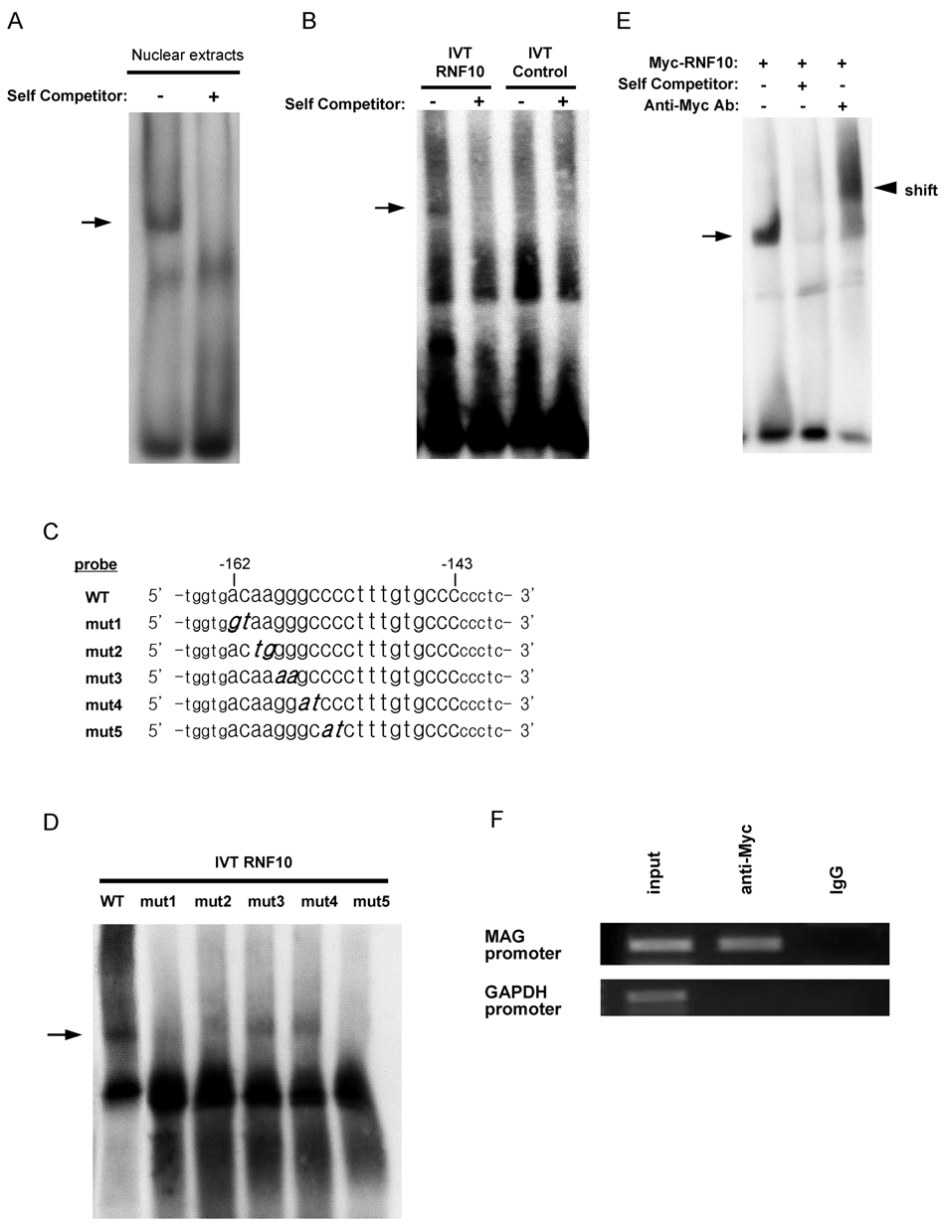
Schwann cell nuclear extract and synthesized RNF10 can bind to the 20-bp *MAG* promoter *cis*-element (−162/−143) designated as “SSE.” (A) EMSA for the binding of nuclear factors. The binding of Schwann cell nuclear extracts (10 µg) with the DIG-labeled *MAG* promoter fragment (−167/−138) was tested in the absence of a specific competitor (lane 1) and in the presence of a 100-fold molar excess of the unlabeled fragment (lane 2). The arrow indicates DNA-protein complexes. (B) RNF10 was in vitro translated from RNF10-pCITE4 plasmid. DNA binding activity of RNF10 was analyzed using DIG-labeled *MAG* promoter oligonucleotide as probe. Translation reaction products with empty vector pCITE4 were used as control. 100-fold molar excess of the unlabeled fragment was used as competitor. Arrows denote the positions of the DNA–RNF10 complex. RNF10 is able to form specific DNA-protein complex with *MAG* promoter oligonucleotide containing “SSE” sequence. (C) Serial 20-bp mutant oligonucleotides of the −162/−153 region of the *MAG* promoter *cis*-element were generated. The flanking sequences are indicated in small typeface, and mutated bases are indicated in italics and bold. (D) EMSA using DIG-labeled mutant oligonucleotides and in vitro-translated RNF10. Any mutation abrogated DNA-protein complex formation in varying degrees. The arrow indicates the DNA-RNF10 complexes. (E) EMSA-supershift analysis of Myc-RNF10. Myc epitope-tagged RNF10 was expressed in Schwann cells by retrovirus-mediated gene transfer. Nuclear extracts were used for the EMSA with a WT probe. DNA-protein complex was observed (arrow). Anti-Myc antibody was added for supershift analysis. The presence of the anti-Myc antibody created the supershift band (arrowhead). (F) Myc-RNF10 was expressed in Schwann cells by retroviral transduction. ChIP assay followed by PCR analyses was performed on Myc-tagged RNF10. A portion of the rat Gap*dh* promoter region that does not contain “SSE”-like sequence was amplified to control for specificity. Input DNA was obtained from formaldehyde-crosslinked sonicated chromatin without immunoprecipitation. IgG was used as negative control.

To determine whether the identified *cis*-acting element, SSE, can recruit transcription factors to regulate the promoter activity, electrophoretic mobility shift assay (EMSA) was carried out using nuclear extracts from rat Schwann cells. We constructed a double-stranded oligonucleotide spanning the region from −167 to −138 in the *MAG* promoter, including the 20-bp *cis*-acting element (−162 to −143), with flanking sequences at both the ends. The SSE sequence, containing the *cis*-acting element, could form sequence-specific DNA-protein complexes with the nuclear extracts of rat Schwann cells (Fig 2A); this suggested the presence of endogenous transcriptional regulatory factors in Schwann cells.

Since the SSE sequence does not contain previously identified transcription factor-binding sites, we screened a cDNA fusion library, which was generated from rat Schwann cells, by using the SSE sequence as a probe in yeast one-hybrid screening. We identified 10 candidate genes containing partial in-frame sequences, namely, *Wbp1*, *Rnd2*, *Sub1*, *Rbm3*, *Rbm5*, *LOC308081*, *Brp44l*, *Pja1*, *Zdhhc16*, and *RNF10*, which encode proteins that appear to exhibit DNA-binding activity. Luciferase reporter gene assay revealed that among these candidate genes, only *RNF10* activated the *MAG* promoter when overexpressed in Schwann cells. Full-length *RNF10* was subsequently isolated from Schwann cell cDNA by RT-PCR with appropriate primers. The final open reading frame was found to potentially encode an 802-amino acid protein with a molecular mass of 88 kDa. RING finger motif is a variant of zinc finger motif present in a family of proteins, including transcription regulators. RNF10 has been shown to be ubiquitously expressed and has been previously isolated from a mouse brain cDNA library [13].

### RNF10 Directly Activates the *MAG* Promoter in Schwann Cells

To determine whether RNF10 directly binds to the SSE, we synthesized RNF10 by in vitro translation and examined its binding to double-stranded SSE oligonucleotide by EMSA. RNF10 expression plasmids were constructed using vector pCITE4. The EMSA revealed the binding of RNF10 to wild-type SSE oligonucleotide, whereas proteins from in vitro translation reaction with control empty vector pCITE4 did not form a complex with oligonucleotide (Fig 2B). In order to map the RNF10-binding motif within the SSE sequence, we analyzed a series of oligonucleotides in which 2 bases were mutated at a time. Such a condition was employed because the luciferase assay had revealed the importance of the first half of the SSE sequence in transcription. Our analysis revealed that mutation of any 1 pair of bases in the ACAAGGGCCC sequence (Fig 2C) abrogated RNF10 binding in varying degrees (Fig 2D). Furthermore, we performed EMSA supershift analysis. Myc epitope-tagged RNF10 was expressed in Schwann cells by retrovirus-mediated gene transfer. EMSA was performed using nuclear extracts from Schwann cells and anti-Myc antibody. The presence of the anti-Myc antibody created the supershift band (Fig. 2E).

We further performed chromatin immunoprecipitation (ChIP) assays to investigate the association of RNF10 with the SSE in Schwann cells. Myc epitope-tagged RNF10 was expressed in Schwann cells by retrovirus-mediated gene transfer. Sheared chromatin was prepared from these cells, Myc-RNF10 was immunoprecipitated, and the DNA present in the immunoprecipitated material was analyzed by PCR. The binding of Myc-RNF10 to the SSE region of the *MAG* promoter was demonstrated by PCR performed with specific primers. The specificity of Myc-tagged RNF10 association with the MAG promoter regions of the chromatin is supported by the observation that anti-Myc antibodies could not precipitate a 5′ regulatory region of the *Gapdh* gene that does not contain SSE-like sequence (Fig 2F). Taken together, these data suggest that RNF10 binds to the DNA fragment containing the SSE sequence.

**Figure 3:**
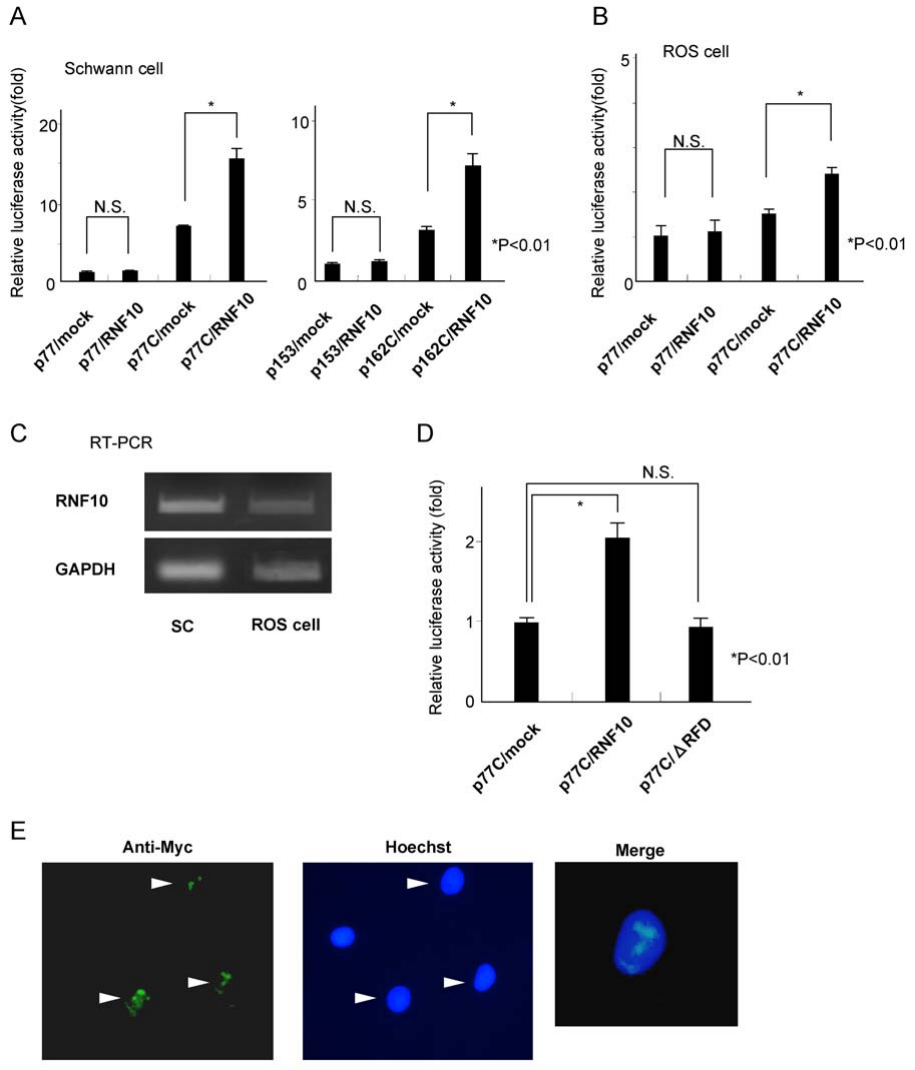
RNF10 activates *MAG* promoter activity in Schwann cells. (A) Schwann cells were cotransfected with an expression construct for RNF10 (pcDNA3.1-RNF10) and *MAG*-promoter-LUC with (p77C, p162C) or without (p77, p153) the tandem SSE region. Overexpression of RNF10 increased the *MAG* promoter activity in Schwann cells by 100% when compared with the activity in the control cells only when the reporter constructs contained the SSE region. Thus, RNF10 increases the promoter activity in a *cis*-element-dependent manner. (B) Cotransfection of an expression construct for RNF10 (pcDNA3.1-RNF10) and *MAG*-promoter-LUC containing the tandem SSE into ROS cells. The *MAG* promoter activity in the ROS cells increased by 60% when compared with the activity in the control cells. However, the degree of activation in ROS cells was not as high as that in the Schwann cells. (C) There was no difference in endogenous RNF10 mRNA expression between SC and ROS cells. (D) Cotransfection of an expression construct for the RING finger domain-deletion mutant of RNF10 (ΔRFD) and *MAG*-promoter-LUC containing the tandem SSE into Schwann cells. The RING finger domain-deletion construct did not activate the *MAG* promoter. (E) Indirect immunofluorescence of Schwann cells indicated the nuclear localization of RNF10. Schwann cells stably expressed Myc epitope-tagged RNF10 by retrovirus-mediated gene transfer. Cells were immunostained with an anti-Myc antibody, and the nucleus was stained with Hoechst 33342. The arrow heads show that tagged RNF10 was almost exclusively located in the nucleus in dot-like structures. The normalized luciferase activity is expressed as fold induction compared with the value of p77/mock or p153/mock in (A), p77/mock in (B) and p77C/mock in (D). *p<0.01; Student's t test. Error bars show mean±SD.

To determine whether RNF10 binds to the M*AG* promoter and enhances promoter activities, Schwann cells were cotransfected with an expression construct for RNF10 (pcDNA3.1-RNF10) and a luciferase reporter construct, p77 or p77C/p153 or p162C (Fig 1C). Overexpression of RNF10 in Schwann cells increased the *MAG* promoter activity by 100%, as compared to the activity in the control cells; however, this was observed only when the reporter constructs contained the SSE region (Fig 3A). We also carried out the same experiments using ROS cells. In ROS cells, overexpression of RNF10 increased the *MAG* promoter activity by 60%, as compared to the activity in the control cells (Fig 3B). However, the degree of promoter activation in ROS cells was not as high as that in Schwann cells. Since no difference of endogenous RNF10 mRNA expression was detected between SC and ROS cells By RT-PCR (Fig 3C), these results suggest that RNF10 can activate the *MAG* promoter more effectively in Schwann cells than in ROS cells.

Furthermore, to examine the role of the RING finger domain, Schwann cells were cotransfected with the vector expressing the RING finger domain deletion mutant of RNF10 (ΔRFD) and the luciferase reporter construct p77C. The ΔRFD construct did not activate the *MAG* promoter (Fig 3D). Taken together, RNF10 directly binds to the *MAG* promoter and enhances promoter activities, and the RING finger domain is required for this interaction.

In order to induce the transcriptional control activity, transcription factors must enter the nuclear compartment. RNF10 protein contains 3 nuclear localization signals in the 592–600 aa, 605–610 aa, and 785–793 aa regions [13]; hence, it would appear that the RNF10 protein is localized in the nucleus. We confirmed the nuclear localization of RNF10 by indirect immunofluorescence (Fig 3E). Myc epitope-tagged RNF10 was expressed by retrovirus-mediated gene transfer. Tag immunostaining showed nuclear staining.

### Specific Silencing of the *RNF10* Gene by RNF10 Small Interfering RNA in Schwann Cells

**Figure 4:**
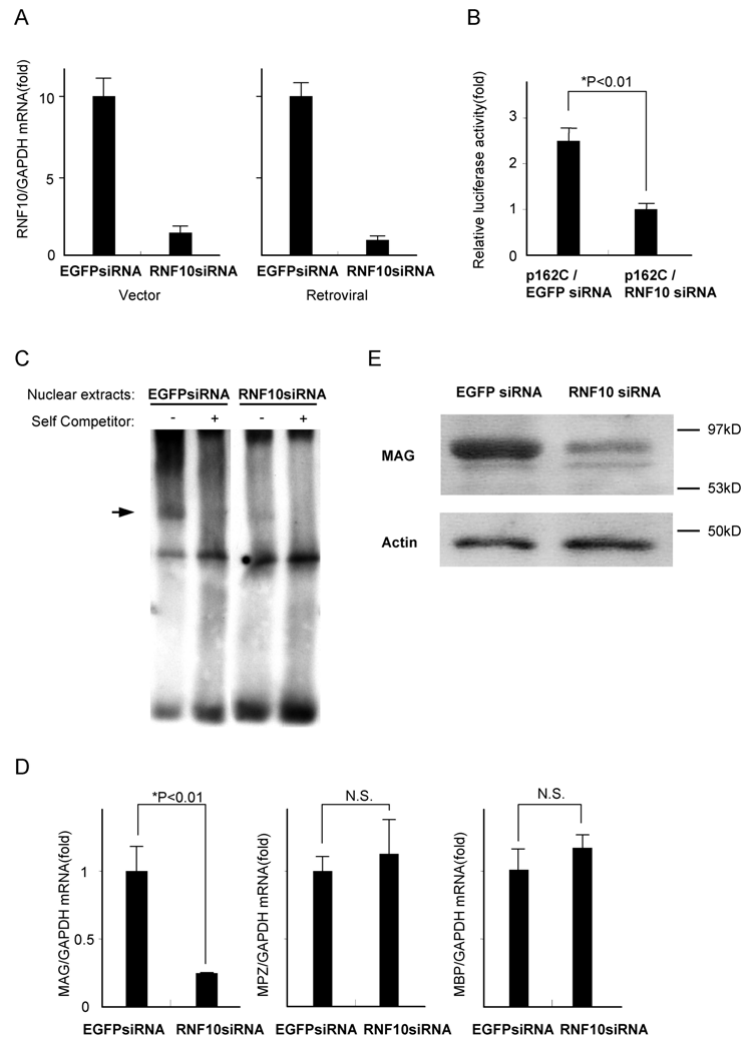
Specific silencing of the *RNF10* gene with RNF10 siRNA in Schwann cells. (A) Schwann cells were transfected with either RNF10 siRNA or control EGFP siRNA expression vector. At 48 h after transfection, quantitative RT-PCR analysis showed that the relative *RNF10* mRNA levels in the RNF10 siRNA-treated cells had reduced to 10% of that in the control EGFP siRNA-treated cells (left). Retroviral siRNA showed approximately the same results (right). (B) Downregulation of *MAG* promoter activity by RNF10 siRNA. Schwann cells were cotransfected with a *MAG*-promoter-LUC containing tandem SSE and RNF10 vector or control EGFP siRNA expression vector. Luciferase activity was measured 48 h posttransfection. RNF10 siRNA suppressed the promoter activity to 40% of that in the control cells. (C) EMSA using Retrovirus-mediated RNF10 siRNA Schwann cells. Schwann cells were stably transfected with a retrovirus-based RNF10 siRNA and control EGFP siRNA. Nuclear extracts were used for the EMSA with a WT probe. DNA-protein complex was observed with the control nuclear extract (arrow), while the nuclear proteins of RNF10 siRNA Schwann cells could not form a DNA-protein complex. (D) Schwann cells were infected with a retrovirus expressing RNF10 siRNA or control EGFP siRNA, selected in puromycin, and analyzed for the expression of MAG by quantitative RT-PCR analysis. Retrovirus-mediated RNF10 siRNA specifically reduced the MAG mRNA expression levels to 25% of that in the control. No difference was observed in the mRNA levels of MPZ and MBP between the RNF10 siRNA-treated and control cells. (E) Western blotting using an anti-MAG or anti-actin antibody of Schwann cells infected with either control EGFP siRNA or RNF10 siRNA-expressing retrovirus. RNF10 siRNA markedly reduced the MAG protein expression in Schwann cells. *p<0.01; Student's t test. Error bars show mean±SD.

To investigate the physiological role of RNF10 in Schwann cells, we interfered in its function by using the small interfering RNA (siRNA). Transfection of the siRNA expression vector into Schwann cells reduced RNF10 mRNA expression to 10% of that in the control cells (Fig 4A). To determine the luciferase activity, the tandem SSE containing the luciferase reporter construct p162C was cotransfected with either RNF10 siRNA or control EGFP siRNA expression vector into Schwann cells. RNF10 knockdown exhibited a significant decrease in the *MAG* promoter activity; the luciferase activity in the RNF10 siRNA-transfected cells was 40% of that in the EGFP siRNA-transfected cells (Fig 4B).

Subsequently, Schwann cells were stably transfected with a retrovirus-based RNF10 siRNA. The RNF10 mRNA levels in the retroviral siRNA-transfected cells were reduced to 10% compared with the control EGFP siRNA- transfected cells (Fig 4A). Then, we performed EMSA analysis using nuclear extracts prepared from Schwann cells transfected with a retroviral RNF10 siRNA and control EGFP siRNA. The nuclear extracts of RNF10 siRNA-treated Schwann cells were not able to form a DNA-protein complex with SSE-containing DNA probe in gel shifts (Fig. 4C). This result further confirmed the specificity of RNF10 in forming complex with SSE sequence.

Using quantitative RT-PCR and western blotting, we next measured the MAG mRNA and protein expression levels in the cells infected with RNF10 siRNA-expressing retrovirus. In RT-PCR, RNF10 siRNA reduced the MAG mRNA levels to 25% of that in the control EGFP siRNA-treated cells (Fig 4D). Western blotting showed that the RNF10 siRNA markedly reduced the MAG protein expression level, when compared with the level in the control EGFP siRNA-treated cells (Fig 4E). We also measured the mRNA levels of myelin protein zero (MPZ/P0) and myelin basic protein (MBP). No difference was observed in the mRNA levels of MPZ and MBP between the RNF10 siRNA-treated and control cells (Fig 4D). This suggests that RNF10 specifically regulates *MAG* gene expression and does not influence the expression of other Schwann cell differentiation markers.

### RNF10 Negatively Regulates Schwann Cell Proliferation

**Figure 5:**
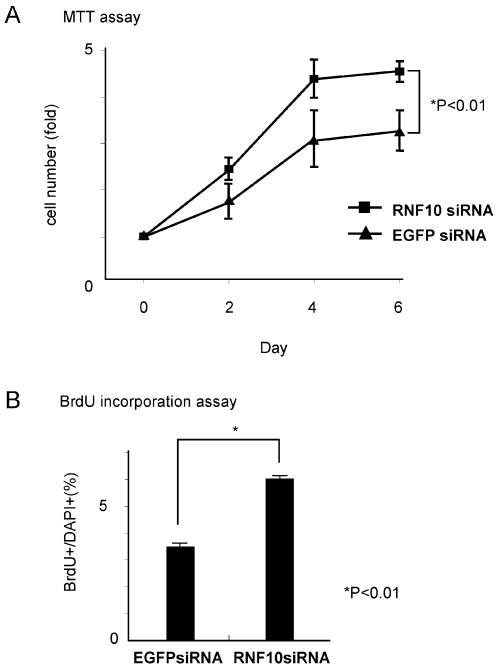
RNF10 negatively regulates Schwann cell proliferation. (A)The time course of cell number was assessed for Schwann cells infected with retrovirus expressing RNF10 siRNA, or control EGFP siRNA by using the MTT assay. Compared to control cells, the *RNF10*-knockdown Schwann cells exhibited a 40% greater increase. (B) BrdU incorporation was significantly increased (80% greater) in stable RNF10 knockdown cells compared with controls. *p<0.01; Student's t test. Error bars show mean±SD.

In addition, we examined the effect of RNF10 on the cell cycle regulation in Schwann cells. In MTT assay, compared to control cells, the number of stable RNF10 knockdown cells exhibited 40% greater increase on day 4 (Fig 5A). To determine whether the increase of cell number was caused by the promotion of cell cycle progression, we performed BrdU incorporation experiments. BrdU incorporation was significantly increased (80% greater) in stable RNF10 knockdown cells compared with controls (Fig 5B). These results suggest that RNF10 is involved in Schwann cell proliferation.

### Knockdown of RNF10 in Schwann Cells Inhibits Myelin Formation

Finally, to investigate the potential role of RNF10 in the process of myelination, we cocultured Schwann cells with DRG neurons, which is a well-established in vitro system for studying myelination.

**Figure 6:**
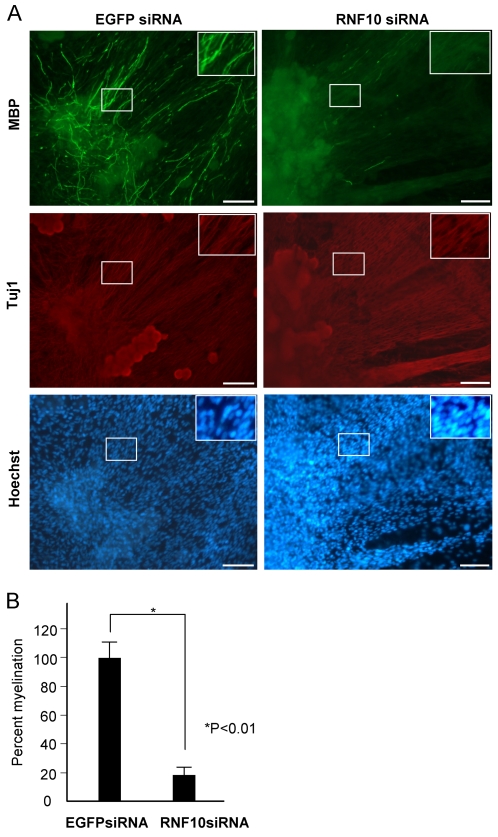
Knockdown of RNF10 in Schwann cells inhibits myelin formation. (A) In vitro myelination using either control EGFP siRNA or RNF10 siRNA retrovirus-infected Schwann cells. Many MBP-positive myelin profiles were detected in cocultures containing the EGFP siRNA-expressing Schwann cells but not in the cocultures containing the RNF10 siRNA-expressing Schwann cells. Similar neurite outgrowth was observed between both cultures. No difference was observed in the cell density between both the cultures. Insets are higher magnification of boxes, respectively. Bar,100 µ. (B) Quantification of myelin profiles in control and RNF10 siRNA panels. Percent myelination value was calculated from 3 independent in vitro myelination experiments.

Schwann cells were stably transfected with a retrovirus-based RNF10 siRNA prior to initiating the coculture and were added to purified DRG neuron cultures. Using this method and retrovirus vectors, we could knockdown the endogenous RNF10 activity in Schwann cells without affecting the neurons. The medium was then replaced with myelin-inducing medium containing ascorbate; after 2 weeks of coculture, the cells were fixed and immunostained with an anti-MBP antibody. Myelin segments were clearly visible in the EGFP siRNA retrovirus-transfected cultures. Compared with the control EGFP siRNA, the RNF10 siRNA resulted in more than 80% reduction in the total number of myelin segments per microscopic visual field. DRG neurons exhibited normal neurite outgrowth in both cultures. There was no difference in cell density between both cultures (Fig 6A, B). This result suggests that RNF10 expression in Schwann cells is indispensable for the Schwann cell differentiation and myelin formation.

## Discussion

In this study, we cloned and sequenced the promoter and 5′ upstream flanking regions of the rat *MAG* gene. The 5′ deletion analysis of the *MAG* promoter revealed that the 20-bp sequence extending from nucleotide −162 to −143 could induce promoter activity at levels similar to that induced by the longest reporter construct. The 20-bp sequence ACAAGGGCCCCTTTGTGCCC (from −162 to −143), which contained no known transcriptional factor-binding sites, was identified as a novel *cis*-acting element and was designated SSE. This sequence has not been identified in previous studies related to the *MAG* gene promoter, probably due to the differences in experimental conditions (we used Schwann cell primary culture instead of the C6 glioma cell line, which has been used in other studies). This sequence is completely conserved in the rat, mouse, and human. We were therefore interested in using the SSE for yeast one-hybrid screening. Consequently, we identified RNF10 as a protein capable of binding to this critical segment of the *MAG* promoter regulatory region. The specificity of this binding was independently confirmed by EMSA and ChIP.

RNF10 is one of the members of the RNF, which has generally been implicated in the development, transcriptional regulation, signal transduction, DNA repair, apoptosis, and oncogenesis [14]. Moreover, *RNF10* is a highly conserved gene, exhibiting 96% homology between rat and mouse and 90% homology between rat and human. Some members of the RNF have been shown to bind with DNA and function as transcriptional activators [15], [16]. RNF10 has been recently shown to associate with Mesenchyme Homeobox 2 (MEOX2) transcription factor, which regulates the proliferation, differentiation, and migration of vascular smooth muscle cells and cardiomyocytes [17]. Although RNF10 has not been investigated in the context of the nervous system, several databases have implicated its expression in the nervous system. We also confirmed the elevation in the RNF10 mRNA level in the rat embryonic PNS (data not shown). Taken together, RNF10 can be considered as a transcription factor participating in the peripheral nerve development and Schwann cell differentiation.

Our data demonstrates that RNF10 binds to the SSE in the *MAG* gene promoter to enhance promoter activities; however, RNF10 may require some partner molecules to perform its function. The existence of such molecules can be predicted based on the findings that the *MAG* gene promoter showed a higher activity in the Schwann cell background compared to that in the ROS cell background. The difference can be attributed to the lack of partner molecules in ROS cells. The precise mechanisms in the RNF, including RNF10, are not completely understood; nonetheless, RNF10 has been reported to form a complex with MEOX2 to regulate transcriptional activities. Therefore, it appears that RNF10 requires a Schwann cell-specific-binding partner to regulate Schwann cell functions. Further studies are required to elucidate such molecules, and these studies would facilitate the understanding of more precise mechanisms underlying RNF10 functions.

Using siRNA, we showed that the loss-of-function of RNF10 in Schwann cells resulted in a decreased MAG expression and reduced myelination in Schwann cell/neuron cocultures. However, in the gain-of-function experiments, the luciferase assay demonstrated that the overexpression of RNF10 in Schwann cells by vector transfection or retrovirus infection caused an increase in the *MAG* promoter activity; however, no detectable difference was observed in myelination when compared with the control cells (data not shown). These results suggest that RNF10 is an essential molecule for initiating myelination in Schwann cells but is not able to promote myelination independently. This interpretation is conceivable because biological processes such as myelin formation involve many steps, and the molecules in each step interact mutually to organize the processes. Therefore, it is intriguing to elucidate the relation between RNF10 and other Schwann cell-related transcriptional factors such as Oct-6 and Krox20. Since MAG expression is induced in the early stages of myelination, we assume that RNF10 participates in the initiation of myelination. Further, it is important to clarify the stage at which RNF10 is indispensable for Schwann cell differentiation by using *RNF10*-deficient mice.

We identified RNF10 as a transcriptional regulator of the *MAG* gene, but we speculate that other target molecules are also regulated by RNF10. Our results indicate that RNF10 is required for both Schwann cell myelination and MAG expression. However, considering that *MAG*-deficient mice are capable of forming the myelin structure with modest abnormalities in the PNS [18], [19], RNF10 probably regulates myelination by not only MAG expression but also other pathways. Some sequences, similar to the SSE, exist in the gene promoter regions of the rat, mouse, and human, suggesting that RNF10 may also regulate other gene expressions. However, we have not yet identified any myelin-related genes near the SSE-like motif region. On the other hand, since the knockdown of RNF10 in Schwann cells induced cell proliferation, we conjecture that RNF10 may be involved in exiting from cell cycle to initiate terminal differentiation and myelination.

In conclusion, we prove that RNF10 binds to the MAG promoter and regulates MAG expression and myelin formation in Schwann cells. RNF10 can be considered as a regulator of Schwann cell differentiation and myelination. These results were obtained by in vitro experiments. The generation of *RNF10*-knockout mice should facilitate the understanding of the in vivo biological function of RNF10.

## Materials and Methods

### Luciferase Reporter Construction and Assay

To generate reporter construct p2752, the −2752 to +78 bp of the gene (+1 bp: transcriptional initiation site shown in reference [20]) of the rat MAG promoter was subcloned into the KpnI, NheI site of pGL3-Basic (Promega). To generate p153 was digested with ApaI and KpnI, ligated, and sequenced. For reporters p283, p162, p77, and p283Δ were generated by PCR with p2752 as the template. Tandem repeats mutation constructs (p162A,B,C) generated by PCR using the primer to which desired sequence was added with p162 as the template. Reporters p77D,C were generated by ligation of oligo insert into upstream of promoter sequence in p77. These constructs were confirmed by sequencing. For luciferase assay, cells were seeded on 24-well plates, cultured overnight, and transfected with the plasmids by using the FuGENE 6 (Roche Biochemicals). Before transfection, Schwann cells were grown in medium containing 2 µM forskolin and 2 nM recombinant neuregulin (rh-HRG-1; Genzyme) and after transfection, cells were incubated in medium with 2 µM forskolin and without neuregulin. The transfected cells were cultured 48 h before harvesting to measure luciferase activity. Cell lysate was assayed by the dual-luciferase (firefly luciferase and control Renilla) reporter assay system from Promega.

### Cell cultures

Primary rat Schwann cells were isolated and cultured using the modified method of Brockers et al. [21], [22]. Briefly, Schwann cells were taken from sciatic nerves of P2 Wistar rats and cultured in DMEM containing 10% FBS. The following day, the medium was supplemented to contain 10 µM AraC to kill dividing fibroblasts for 48 h. For routine culture the cells were grown on poly-L-lysine-coated tissue culture dishes in DMEM containing 10% fetal bovine serum, 2 µM forskolin and 2 nM neuregulin.

### Electrophoretic mobility shift assay (EMSA)

Nuclear extracts from rat primary cultured Schwann cells were prepared using the NE-PER kit as per manufacturer's recommendations (Pierce). RNF10 proteins were in vitro transcribed and translated from RNF10-pCITE4 plasmid using the TnT(R) Quick Coupled Transcription/Translation system (Promega).

The sequences of the oligonucleotide probes used were as Fig 2B. Synthesized double-stranded oligonucleotides were then DIG end-labeled, and EMSA was performed utilizing the DIG gel shift kit as per manufacturer's recommendations (Roche). Binding reactions were performed in the mixture containing nuclear proteins (3 µg) or 2 µl of in-vitro translated products, poly(d(I-C)) (0.5 µg), and the DIG-labeled probe (15 fM) in a final volume of 20 µl of reaction buffer. The complexes were separated on 6% polyacrylamide gels in Tris-borate-EDTA. The gels were blotted onto positively charged nylon membranes (Roche) and developed by an anti-DIG antibody coupled to alkaline phosphatase (Roche). CDP-Star (Roche) was used as substrate for the alkaline phosphatase. Antibody supershift analyses were performed with a monoclonal anti-c-Myc antibody (Clontech). The EMSA binding buffer was used to prepare the working dilution. Nuclear extracts, antibody, and probe were incubated at RT for 45 min before this reaction mixture was loaded on 6% acrylamide gels.

### Yeast one-hybrid screen

Yeast one-hybrid screen was carried out according to the manufacturer's protocol (MATCHMAKER One-Hybrid System; Clontech). Briefly, three tandem copies of the 20 bp cis-acting Element (SSE; 5′- ACAAGGGCCCCTTTGTGCCC -3′) were synthesized, annealed, and cloned into the pHIS2 bait vector. The SMART cDNA Library Construction Kit (Clontech) was used to synthesize cDNA from primary cultured rat Schwann cells. The isolated cDNA and Sma-I linearized pGADT7-Rec2 vector and bait vector were then transformed into yeast strain Y187, and the transformants were selected on SD/-His/-Leu/-Trp medium containing 15 mM 3AT (Sigma). The plasmids from positive yeast colonies were isolated and transformed into DH5α bacterial competent cells and each cDNA insert was sequenced, and BLAST searched.

### Chromatin immunoprecipitation(ChIP) assays

For overexpression experiments, Schwann cells were infected with retrovirus to express Myc epitope-tagged RNF10 and selected by puromycin. ChIP assays were performed using a commercial kit (Upstate Biotechnology) as described by the manufacturer. Briefly, DNA was crosslinked to protein with formaldehyde. Cellular lysates were obtained by scraping, followed by pulsed ultrasonication to shear cellular DNA. For immunoprecipitation, anti-Myc antibody (Upstate Biotechnology) or mouse IgG (negative control) was added overnight at 4°C. On the next day, the crosslinks were reversed, and bound DNA was purified. PCR to detect the SSE region (−162/−143) was performed with the following primers: 5′- TCTCCAGGAAGGAGTTAGTAGGAG-3′, 5′- ACCACTAAGGTCTTTGGTTGGTTT-3′ (−217/+212). Primers amplifying the promoter region of the rat Gapdh gene were used as a negative control (forward, 5′-AAACAAGTTCACCACCATGTGAAA-3′; reverse, 5′-CCAGGGATTGACCAAAGGTGAGTT-3′).

### RNAi

siRNA-expressing plasmid vector driven by mouse U6 promoter (piGENE mU6 vector: iGENE) were constructed according to the the manufacturer's protocol. 62-base DNA oligonucleotides corresponding to sense target sequence, hairpin loop, and antisense target sequence were synthesized (invitrogen), annealed together, and then ligated into piGENE mU6 vector. The oligonucleotides synthesized for RNAi were as follows: 5′-GTTTGGAGAGAATTCAGATGGATTCGTGTGCTGTCCGGATCCATTTGAGTTCTTTCCTTTTT -3′ and 5′- ATGCAAAAAGGAAAGAACTCAAATGGATCCGGACAGCACACGAATCCATCTGAATTCTCTCC -3′ (RNF10) and; 5′-GTTTGGCTATGTCCGGGAGTGCATCGTGTGCTGTCCGGTGCGCTCCTGGACGTAGCCTTTTT -3′ and 5′-ATGCAAAAAGGCTACGTCCAGGAGCGCACCGGACAGCACACGATGCACTCCCGGACATAGCC -3′ (enhanced green fluorescent protein; EGFP).

For stable gene knockdown the pMX–puro (kindly provided by T. Kitamura, University of Tokyo, Japan) vector was used [23]. The mouse U6 gene promoter and shRNA sequence from piGENE mU6 was subcloned into pMX–puro vector, yielding pMX–puro mU6/siRNA. The generated retroviral construct was used to produce recombinant viruses and infect the indicated cells. Infected schwann cells were selected with 1 µg/ml puromycin for 48 h.

### Real-time reverse transcription-PCR

Total RNA was isolated using Isogen (Wako). Total RNA (1 µg) was used in a reverse transcription (RT) reaction with the QuantiTect Reverse Transcription kit (Qiagen), according to the manufacturer's instructions. cDNA was used as template in real-time PCR assays based on SYBR Green detection with the ABI Prism 7000 Sequence Detection system. PCR primers were as follows:

MAG forward, 5′-GACAAGAAGAAAAAAGAACGTCACAGA;

MAG reverse, 5′-AGGCGCTTCTCACTCTCATACTTATCA;

RNF10 forward, 5′-TATTCCATGTCTGAGGATGTTCGACA;

RNF10 reverse, 5′-TCTCAATGTCATCTGAGAACATCTCT;

GAPDH forward, 5′-TCCTGCACCACCAACTGCTTAG;

GAPDH reverse, 5′-GATGACCTTGCCCACAGCCTTG.

### Cell proliferation assay

Cell proliferation was evaluated in 96-well plates by the modified MTT method designated WST-8 as indicated in the manufacture's instruction (Dojindo Laboratories). Briefly, Schwann cells were seeded into 96-well tissue culture plates at a concentration of 3×10^3^ cells/well and incubated in DMEM with 10% FBS and antibiotics at 37°C. After 3 h (day0), 48 h (day2), 72 h (day4), 96 h (day6), the absorbance at the 490-nm wavelength was measured using an automated microplate reader (Bio-Tek Instruments).

BrdU incorporation assays were performed by incubating cells in 24-well culture plates and labeling with 10 µM bromodeoxyuridine (BrdU) in medium for 18 h. Afterward, the cells were fixed for 20 min in 4% paraformaldehyde. For BrdU immunocytochemistry, the cells were then treated with MeOH for 10 min at 4 d, followed by 2 M HCl for 30 minutes at room temperature, and reacted with primary and secondary antibodies using standard protocols. The cells were counterstained with the nuclear stain DAPI. The percentage of cells that had incorporated BrdU was quantified by determining the ratio of BrdU-positive nuclei and the total number of nuclei in 15 systematically sampled microscopic fields.

### Western blotting

Rat Schwann cells on 60 mm plates were lysed in M-Per™ mammalian protein extraction reagent (Pierce) containing a complete™ mini protease inhibitor cocktail tablet (Roche). Samples containing an equal amount of protein (10 µg) were analyzed by SDS-PAGE. Proteins were then transferred to a PVDF membrane (Bio-Rad). Primary antibodies (anti-MAG; MAB1567, Chemicon) were used in 1∶1000 dilution at room temperature for 1 hr in 3%BSA TBS plus Tween 20. The secondary antibodies were used in 1∶10,000 dilution, and the immunoblots were developed by using the ECL system (Amersham Biosciences).

### Myelination assay

DRG neuron coculture with Schwann cells was performed using the modified method of Einheber et al. [24]. Briefly, DRGs were taken from E15 rats, dissociated with trypsin, and seeded on 12 mm dishes coated with collagen type I at 200,000 cells per well. Non-neuronal cells were removed by treating cultures with C media (MEM, 10% FBS, 0.4% glucose, and 100 ng/ml 2.5S NGF) supplemented with uridine, and AraC (at 10 µM) or with C media alone every 2–3 d alternately for 10 d. For myelination, 250,000 retroviral-infected Schwann cells were seeded onto DRG cultures in DMEM/F12 medium with N2 supplement (Gibco) and 50 ng/ml NGF and kept for several days until axons were populated by Schwann cells. The medium was then switched to C media with 50 ng/ml ascorbic acid to promote myelination. Cocultures were kept for 2 weeks and then fixed with 4% paraformaldehyde, and anti-MBP and anti-Tuj1 antibody diluted at 1∶200 in TBST with 3% BSA was applied overnight at room temperature. Secondary antibodies were then added to observe myelin-forming Schwann cells by fluorescence microscopy. The number of myelin-forming Schwann cells were counted by surveying 20 fields at 200_magnification from three independent experiments and expressed by percentage ratio to that in control cultures. The average was calculated, and *p*- values were obtained using Student's *t*- test.
